# Surgical Conundrum: A Case of Giant Renal Angiomyolipoma Abutting Inferior Vena Cava With Haemorrhage

**DOI:** 10.7759/cureus.30016

**Published:** 2022-10-07

**Authors:** Sweta Sahu, Roopeessh Vempati, Vaidehi Mendpara, Megha Yadav, Anam Sayed Mushir Ali, Anubhuti Vashyani, Mummareddi Dinesh Eshwar, Dharmesh R Chauhan

**Affiliations:** 1 Surgery, Jagadguru Jayadeva Murugarajendra (JJM) Medical College, Davanagere, IND; 2 Internal Medicine, Gandhi Medical College & Hospital, Hyderabad, IND; 3 Medicine and Surgery, Government Medical College, Surat, IND; 4 General Surgery, Maharani Laxmi Bai Medical College, Jhansi, IND; 5 Medicine, Indian Institute of Medical Science and Research, Aurangabad, IND; 6 General Surgery, Indira Gandhi Medical College and Hospital, Shimla, IND; 7 Biochemistry, Mahavir Institute Of Medical Sciences, Hyderabad, IND; 8 General Surgery, Government Medical College, Surat, IND

**Keywords:** inferior vena cava, angiomyolipoma, surgery, giant renal aml, ivc abutting, nephrectomy, renal angiomyolipoma

## Abstract

Renal angiomyolipoma (AML) is not only uncommon but often an accidental diagnosis, as it is frequently asymptomatic and affects females disproportionately. Although they may exhibit symptoms of tuberous sclerosis complex and lymphangioleiomyomatosis, the vast majority are sporadic. Due to its vascular nature, AML is prone to bleeding, and AML that bleeds typically belongs to the tuberous sclerosis complex. AMLs are mostly benign, but they can proliferate and invade locally, necessitating a strict management strategy. We hereby delineate the manifestations of a 32-year-old man who complained of increased abdominal size and pain without any history of injury. On examination, abdominal distension was demonstrated, and a palpable mass was detected in the right hypochondrium and right lumbar area. All findings from various diagnostic methods indicated that it was a classic kind of renal AML. We are keeping track of this case because it is rare and quite uncommon in males. The case presented a challenging time for the surgeons to plan the line of treatment.

## Introduction

Renal angiomyolipoma (AML) of the kidney is a mesodermal tumour made up of adipose tissue, smooth muscle, and blood vessels. It accounts for 1-3% of solid renal cancers. Pain in the flank, the presence of a palpable mass, and blood in the urine are considered to be the hallmark symptoms of AML. The most serious complication associated with it is the possibility of spontaneous bleeding in the retroperitoneum or the renal collecting system, which might put the patient in a life-threatening condition [1]. It happens to be benign in nature and rare in variety. Approximately 80% of these cancers are sporadic. In certain instances, however, they have been linked to the tuberous sclerosis complex and most commonly affect females [2]. AML is likewise known as renal hamartoma. Previous research suggested that the maximum size of renal AML could grow by 4 cm per year [3,4]. Renal AMLs are referred to as "giant" AMLs when they exceed 10 cm in size. A very small number of giant renal AMLs have been documented in the literature [5]. The haemorrhagic aneurysms that develop inside the AML increase the chances of compression symptoms, and they may rupture and cause bleeding [6]. Depending on the size of the tumour, the expansion of a tumour into the inferior vena cava is frequently observed in renal cell carcinoma but can also occur rarely in AMLs [1].

## Case presentation

Case summary

A 32-year-old man with no previous history of trauma arrived at our hospital's outpatient department complaining of abdominal distension and discomfort. During clinical examination, the patient's pallor, abdominal distension, and a palpable lump in the right hypochondrium and lumbar area were all noted. An ultrasonographic scan revealed a 20 × 11 cm lesion with a broad, well-defined heterogeneous echo texture, along with indications of several hypoechoic regions. The right suprarenal area had few blood vessels inside, which pushed the top of the right kidney down.

On contrast-enhanced computed tomography (CECT) of the abdomen, a well-defined soft tissue density lesion was noted in the right suprarenal region with an ill-defined posterior wall (approximately 12.8 × 13.6 × 17.9 cm), consisting of a hyperdense component (HU +56) suggestive of haemorrhage and a fat density component. The lesion had a preserved fat plane and ran down the inferior vena cava and the underside of the right lobe of the liver. The right kidney was displaced downward because of the lesion. The right adrenal gland was obscured by the lesion and was otherwise inconspicuous.

Pre-operative management

The results of a laboratory examination of the patient showed that the patient had high levels of mean glucose and low levels of haematocrit and haemoglobin. Additionally, the glycated haemoglobin (HbA1C) was high (Table 1). Initially, anaemia was corrected with five pints of packed red cell transfusion. Post-transfusion haemoglobin was 10.1 g/dl. The patient was also diagnosed to be diabetic de novo and adequate glycaemic control was achieved prior to surgery.

**Table 1:** Pre-operative investigations AST: aspartate transaminase; ALT: alanine transaminase; ALP: alkaline phosphatase; ALB: albumin; HIV: human immunodeficiency virus; ECG: echocardiogram; PA: posteroanterior; HbA1C: glycated haemoglobin.

Parameter	Patient’s report	Normal range	Comment
Haemoglobin (g/dl)	5.1	13.5-17.5	Low
Platelet count (lakh per mm3)	4.2	1.5-4.5	Normal
Total leucocyte count (per mm3)	7,500	4,500-11,000	Normal
Haematocrit (%)	16.2	37-53	Low
Serum creatinine (mg/dl)	0.9	0.7-1.18	Normal
Serum urea (mg/dl)	20	17-43	Normal
AST (U/L)	20	<50	Normal
ALT (U/L)	23	<50	Normal
ALP (U/L)	45	38-126	Normal
Serum ALB (g/dl)	4.2	3.5-5.2	Normal
Serum globulin (g/dl)	2.5	2.5-3.5	Normal
Total protein (g/dl)	6.8	6.6-8.3	Normal
Total bilirubin (mg/dl)	1.2	0.3-1.2	Normal
Direct bilirubin (mg/dl)	0.16	<0.2	Normal
Serum sodium (mEq/L)	138	136-146	Normal
Serum potassium (mEq/L)	4.2	3.6-5.2	Normal
Serum chloride (mEq/L)	99	96-106	Normal
Mean blood glucose (mg/dl)	230	70-99	High
HbA1C (%)	8.29	5.7	High
HIV 1 and 2	Non-reactive		
Hepatitis B	Non-reactive		
Hepatitis C	Non-reactive		
ECG	Within normal limits		
Two-dimensional echocardiography	Normal study with an ejection fraction of 60%		
Chest X-ray PA view	Within normal limits		

Surgical management

**Figure 1:**
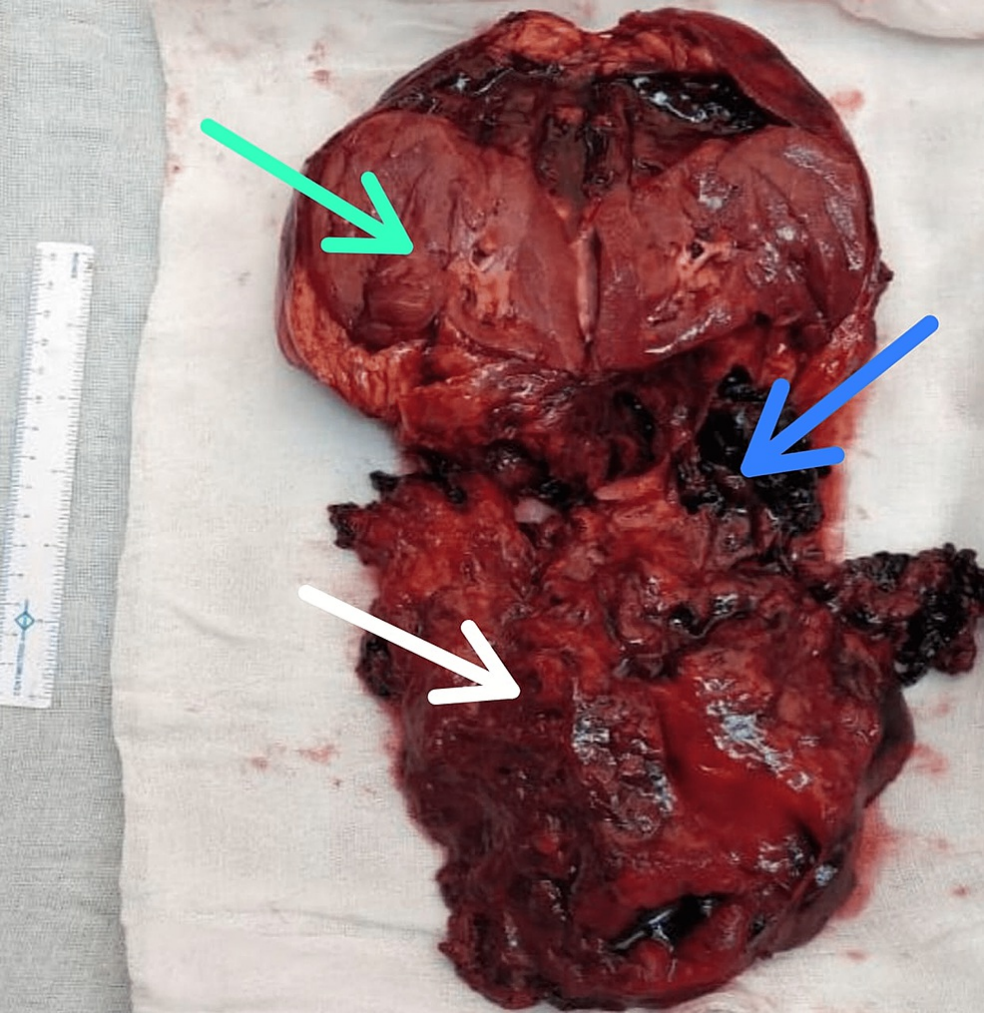
Soft tissue specimen of right kidney with en mass and haematoma The green arrow marks the kidney, the white arrow shows the tumour, and the blue arrow shows the haemorrhagic area.

Exploratory laparotomy with right-side nephrectomy was performed. En masse, with large haematomas, was also removed. The resected mass was then sent for further examination (Figure 1). The post-operative period was uneventful and the patient was discharged without any complications.

Histopathological findings and immunochemistry

**Figure 2:**
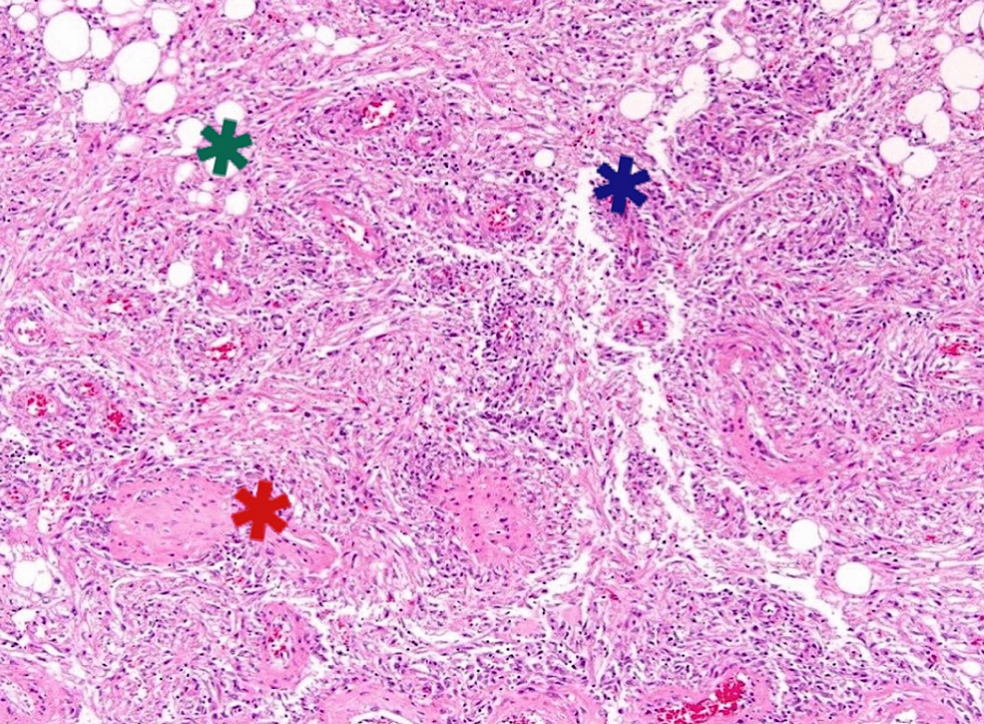
Histopathological view of the tumour The mature adipose tissue is shown by green asterisks, thickened blood vessels are indicated by red asterisks, and smooth muscle is indicated by blue asterisks.

On histopathological examination, the mass showed a variable mixture of predominantly mature adipocytes along with thick-walled vessels. A few foci showed the proliferation of smooth muscle cells around blood vessels, which were epithelioid/spindle in shape, having round to oval nuclei, inconspicuous to prominent nucleoli, fine chromatin, and moderate cytoplasm, which was suggestive of a classic variant of renal AML (Figure 2). Immunohistochemistry reports revealed that the mass was immunoreactive for smooth muscle actin (SMA) and vimentin. The mass was focally immunoreactive for HMB45. All findings were suggestive of AML of the right kidney.

## Discussion

Renal AML, a tumour made up of mesenchymal cells that are harmless, was first talked about in 1951 [7]. A contemporary classification system for renal AML has recently been put into use in clinical practice. This classification divides AMLs into three groups: those with lots of fat, those with little fat, and those with no fat at all. The backbone of this division is the quantitative criteria of chest tomography and magnetic resonance imaging [8]. Retroperitoneal haemorrhage, which is also known as Wunderlich syndrome, causes lower back pain, blood in the urine, and shock. Most of the time, these symptoms lead people with renal AML to see a doctor. AML rupture is caused by factors related to the tumour, such as intratumor aneurysms that are larger than 4 cm, those that are linked to tuberous sclerosis complex or lymphangioleiomyomatosis, and those that are larger than 5 mm [9,10]. As a result of physiological changes that lead to tumour and aneurysm growth, pregnancy also raises the risk of rupture [11]. AML rupture is also at risk from non-tumour-related causes, including coagulopathy and coronavirus disease 2019 (COVID-19) [11]. Since the great majority of tumours with a diameter of less than 4 cm are asymptomatic, individuals with these tumours can be kept stable or treated with radiofrequency ablation [12,13]. To reduce tumour size, immunomodulation with mammalian target of rapamycin (mTOR) inhibitors slows vascular epithelial development [14].

The major treatment option for bleeding AML is often renal artery embolization, which can also be used as a preventative measure for tumours that are 4-10 cm in size [15,16]. For tumours bigger than 4 cm, especially big ones that keep bleeding or look like they might be cancerous, a partial or complete nephrectomy is best [17]. Since the patient in our case did not have a relevant family history, the condition was thought to be random. The need for medical treatment was delayed due to persistent bleeding and haematoma development, which also resulted in significant anaemia that required to be addressed before surgery. Because of the tumour's size and the fact that it was encroaching on nearby tissues and bleeding spontaneously, a total nephrectomy was performed along with the tumour en masse with haematoma.

## Conclusions

A solid understanding of radiological appearance, classifications, and kinds helped us in the accurate diagnosis of the condition. Accurate and quick diagnosis established management strategies for our patient. Our diagnosis was supported by immunohistochemistry and histopathology. To lower the danger of bleeding and ease the compression brought on by this tumour's high risk of bleeding, nephrectomy was the preferred therapy option in our case, as compared to a cautious strategy. At discharge, the patient was instructed to take special care of his wounds, to eat a high-protein diet, and to strictly follow his anti-diabetic drug regimen with frequent glucose monitoring and follow-up visits to his diabetologist.
